# Gait pattern in patients with peripheral artery disease

**DOI:** 10.1186/s12877-018-0727-1

**Published:** 2018-02-20

**Authors:** Maria Szymczak, Paweł Krupa, Grzegorz Oszkinis, Marian Majchrzycki

**Affiliations:** 1 Clinic of Rheumatology and Rehabilitation, Poznan University of Medical Sciences, Poznań, Poland; 2 Faculty of Physical Education, Sport and Rehabilitation, E. Piasecki Academy of Physical Education, Poznań, Poland; 3 Clinic of General and Vascular Surgery, Poznan University of Medical Sciences, Poznań, Poland

**Keywords:** Intermittent claudication, Lower limb ischaemia, Gait pattern

## Abstract

**Background:**

The aim of the present paper is to assess the gait pattern of patients with Peripheral Artery Disease (PAD). A more specific aim is to compare the gait pattern of PAD patients before and after the appearance of intermittent claudication symptoms.

**Methods:**

The study involved 34 PAD patients with a claudication distance ≥200 m and 20 participants without PAD, who formed the control group. The gait pattern of PAD patients was assessed twice: before the appearance of intermittent claudication symptoms (pain-free conditions) and after the appearance of intermittent claudication symptoms (pain conditions).

**Results:**

Compared to the control group, PAD patients presented a statistically significant decrease in step length both during pain-free conditions (52.6 ± 12.5 vs. 72.8 ± 18.5 cm, *p* = 0.008) and in pain conditions (53.3 ± 13.3 vs. 72.8 ± 18.5 cm, *p* = 0.006). As for the remaining spatiotemporal parameters, there were no differences observed between the patient group and the controls. Intermittent claudication symptom induced by the walking test on the treadmill did not bring about any new abnormalities in the gait pattern or intensify the existing abnormalities of the gait.

**Conclusions:**

PAD patients have a tendency to shorten their step length regardless of the presence of intermittent claudication.

## Background

The most frequent symptom of PAD (Peripheral Artery Disease) is intermittent claudication. It manifests itself through pain occurring in one or both lower limbs during physical exercise, and its growing intensity forces the patient to rest. The pain in lower limb muscles is connected with temporary ischaemia caused by arterial occlusion or stenosis. The location and extent of arteriosclerotic lesions determine the location of pain in the lower limbs experienced by the patient [1–3].

Currently, it is believed that PAD patients move more slowly and tend to take shorter steps compared to the healthy population [4, 5]. It is also believed that in the course of PAD, the proportional share of particular phases of the gait cycle is altered. The stance phase is extended, while the swing phase is reduced [6]. In the case of symptoms diagnosed only in one lower limb, the stance phase is reduced for the lower limb affected in order to reduce its load, thus extending the stance phase for the unaffected or less affected limb [7]. Gait abnormalities result in an increase in energy expenditure, deterioration of gait economy [8] and increased risk of falling [9–11].

Gait abnormalities in PAD patients have not yet been explicitly explained. Chen et al. suggest that these abnormalities may be a consequence of weakened back thigh muscles and shin muscles. According to their study, the weakening of these muscles affects the acetabulofemoralor hip joint and ankle mobility, resulting in a slower and heavier gait [12]. The loss of strength and endurance of lower limb muscles stems from histological and metabolic changes. Patients with advanced PAD suffer from atrophy of muscle fibers, which are replaced by connective tissue [13, 14]. One of the underlying causes of that process is the increase in the expression of the transcription factor NF-kB – a protein complex that stimulates catabolic activity in muscles [15]. The progress of the disease is probably related to a gradual proportional decrease in the number of fast-twitch fibers with a high oxygen potential and an increase in the proportion of slow-twitch fibers with a low oxygen potential [13]. Metabolic changes in lower limb muscles are caused by the deteriorated activity of mitochondrial enzymes, such as cytochrome oxidase, succinic acid oxidase, and citric acid synthesis [16–18]. The increasing load from physical effort disturbs the balance between the amount of oxygen supplied and the demand for oxygen. Oxygen synthesis of ATP becomes inefficient, which triggers oxygen-free processes [13].

A controversial issue regarding the gait of PAD patients is the moment at which the pattern of gait is altered. According to some authors, gait abnormalities in PAD patients can be observed both during pain-free and pain conditions, and the appearance of intermittent claudication symptoms only intensifies the existing pathology [6, 12]. Other researchers suggest that gait abnormalities in PAD patients were observed only after the symptoms of intermittent claudication had appeared, while prior to that moment, the gait pattern remained normal [7, 8, 19, 20]. The main aim of the present work was to assess the gait pattern of PAD patients. A more specific aim was to assess the spatiotemporal parameters of the gait of PAD patients before and after the appearance of intermittent claudication symptoms.

## Methods

### Participants

The study involved 54 subjects aged 55–65. Thirty-four participants were PAD patients (stage IIa of the disease according to the Fontaine classification) treated at the Clinic of General and Vascular Surgery, Karol Marcinkowski University of Medical Sciences, Poznań. The patients qualified for the research included those with one-sided and two-sided symptoms. The control group consisted of 20 participants without PAD, whose age was similar to that of the members of the patient group. The inclusion criteria for the control group were: lack of pain in lower limbs and no history of ruptures or surgical interventions. The control group was recruited from among participants of courses at the University of the Third Age in Poznań.

### Inclusion criteria

The following inclusion criteria were adopted: exertional pain in the lower limbs reported by the patient and confirmed in a diagnostic walking test, walking distance ≥ 200 m, resting Ankle Brachial Index (ABI) ≤ 0.9, age 55–65, and written informed consent to participate in the study.

### Exclusion criteria

Conditions regarded as criteria excluding the patient from the research included: a symptom-free course of PAD; walking distance < 200 m; the technical inability to assess ABI; the inability to walk on the treadmill; diabetes; joint disorders obstructing motion; amputation of a lower limb; ulceration of the lower limbs; a stroke suffered within 6 months prior to the study; acute coronary syndrome, coronary artery bypass surgery or endovascular angioplasty of coronary arteries within 12 months prior to the study; surgical or endovascular intervention in the lower limbs within 12 months prior to the study; aortic aneurysm; advanced kidney or liver failure, failure of the respiratory or circulatory system; active neoplastic disease; advanced, chronic venous insufficiency; Buerger’s disease; uncontrolled hypertension; obesity (BMI > 30 kg/m^2^); mental disease or dementia obstructing contact with the patient and simultaneous participation in other clinical trials.

### Early qualification

PAD was diagnosed on the basis of patient interviews and specialized tests including arterial pulse examination in superficial femoral, popliteal, posterior tibial, dorsalis pedis arteries. In order to confirm the early diagnosis, ABI was measured. To visualize the changes in lower limb arteries, patients with ABI < 0.9 were subjected to imaging tests. The tests were carried out by a vascular surgeon.

### Walking distance measurements

The walking tests were performed on a KettlerTrack Motion 7881–300 treadmill (Kettler, Germany) with the use of the Gardner protocol with a gradually increased grading. The treadmill velocity was 3.2 km/h and remained constant. During the first two minutes, the patients walked on a flat surface, without any grading of the treadmill. The grading was increased by 2% every two minutes until the maximum grading of 12% was reached. For each patient, the pain-free walking distance was measured, as well as the maximum claudication distance.

### Assessing the gait pattern

The assessment of spatiotemporal parameters of gait was performed with an optical system of movement analysis Optogait (Microgate, Italy) [21–23]. The measurement unit included two bars: a transmitting bar and a receiving bar, which were positioned parallel to each other on the sides of the treadmill. Each bar contained 100 LEDs (light-emitting diodes) positioned at 1-cm intervals. The LEDs on the transmitting bar communicated on an infrared (visible) frequency with the LEDs on the opposite bar. The system detected the interruptions of the communication between the bars and calculated their duration. The gait pattern test was performed without any grade on the treadmill. The participants walked on the treadmill with a preferred speed, which they believed corresponded best to their daily walking routine.

Bearing in mind that walking on a treadmill is not a natural way of moving for humans, each participant was familiarized with this type of walking prior to the gait analysis. Following the guidelines set out by Mika et al., the gait analysis was preceded by a six-minute warm-up on the treadmill. The study by Mika et al. showed that changes in the gait biomechanics occur during 6 min of walking on a treadmill. After this time, the differences between repeated measurements of specific gait parameters cease to be statistically significant [24]. In our study, sick patients who were unable to walk on the treadmill without a pause were allowed to stop and the durations were aggregated. After becoming familiar with walking on the treadmill, the participants rested in a sitting position for 30 min or longer until the feeling of fatigue/pain in the lower limbs had ceased completely.

The gait pattern in pain-free conditions was analyzed first. Participants reported the moment when the first symptoms of intermittent claudication appeared, which was recorded in the test report and was treated as completing the first stage of the test. The second gait pattern analysis was performed when the ischemic pain reached moderate intensity, assessed by the participant as level 4 pain on a 5-point scale of claudication (where 1 indicates no pain, 2 – the occurrence of pain, 3 – light pain, 4 – moderate pain, 5 – maximum intensity of pain). In the control group, the gait pattern examination continued for 10 min. Members of the control group walked on the treadmill at their preferred speed.

The following spatiotemporal parameters of gait were assessed:

- the proportional share of the swing and stance phases,
- the proportional share of the single support phase and double support phase,
- walking speed [m/s],
- step length [cm],
- cadence,
- gait cycle duration [s].

### WIQ questionnaire

The assessment of the subjective functional capabilities connected with gait was performed with the use of the Walking Impairment Questionnaire (WIQ). The following three domains were assessed: claudication distance, walking speed, and the ability to go up and down stairs. The scoring for each question featured a scale from 0 (the worst possible result of the functional condition measurement) to 4 (the best functional condition) [25].

### Statistics

Statistical analysis was performed with the Shapiro-Wilk test. The Mann-Whitney test was used to assess the parameters for which a non-parametric distribution was obtained. The Wilcoxon signed-rank test was used to compare non-parametric dependent variables. In the case of parameters for which the distribution was close to normal, Student’s t-test was used for the dependent and independent variables. The results obtained by the patient group were compared with the results obtained by the control group (I vs. III and II vs. III, Table 2). Then, the results obtained by the patient group prior to the appearance of intermittent claudication symptoms were compared with the results obtained by the same group after the appearance of intermittent claudication symptoms (I vs. II, Table 2).

## Results

Fifty-four candidates were finally selected to participate in the research. Thirty-four of them were PAD patients. Twenty candidates without PAD were enrolled into the control group. All participants were Caucasian. The characteristics of both groups are presented in Table 1. The average duration of PAD in the patient group was 72.7 ± 20.3 months. The average distance after which the participants started to experience pain in the lower limbs was 254.1 ± 69.9 m. The average maximum claudication distance forcing the patients to stop walking was 384.7 ± 129.8 m. From the anatomical point of view, the patients presented various forms of lower-limb ischaemia: femoral-popliteal PAD in 16 patients, multilevel PAD in 10 patients and aortoiliac PAD in 8 patients. In the case of 16 patients, lesions were located in the right lower limb, in the case of 10 patients – in the left lower limb, and in the remaining 8 patients – in both lower limbs.

**Table 1:** The characteristics of both groups analyzed

Parameter		Patients with PAD	Control group
Age (years)		64.2 ± 5.7	62.7 ± 6.65
Gender	Women	22	12
	Men	12	8
Body Mass Index		22.0 ± 2.5	22.4 ± 2.6
Ankle Brachial Index		0.63 ± 0.09	1.0 ± 0.06
Walking Impairment Questionnaire	Distance	45.4 ± 21.6	96.81 ± 5.14
	Walking speed	38.2 ± 22.9	93.48 ± 5.61
	Stairs	40.2 ± 26.0	96.67 ± 7.30
Smoking	Currently	14	8
	In the past	10	0
	Non-smokers	10	12

*PAD* Peripheral Artery Disease

Spatiotemporal parameters of gait for both groups are presented in Table 2. A comparison of the patient group and the control group indicates that a statistically significant difference was obtained only for one spatiotemporal parameter: a step length. Compared to the control group, PAD patients took shorter steps both before and after the appearance of claudication symptoms. This resulted in an increase in the number of gait cycles performed within 60 s. Moreover, PAD patients presented an increased cadence of gait, a decreased walking speed, and a shorter duration of the entire gait cycle. However, the groups did not differ significantly with regard to these parameters. No statistically significant differences were observed for the length of particular phases of the gait cycle between the groups.

**Table 2:** Gait pattern analysis

Comparison	I. Patients with PAD - before pain	II. Patients with PAD - after pain	III. Control group - no pain	*P* value		
				I vs. III	II vs. III	I vs. II
Support phase overall [%]	62.5 ± 6.6	62.7 ± 6.0	62.2 ± 6.8	0.893^T^	0.830^T^	0.801^T^
Support phase – affected limb [%]	62.7 ± 6.6	63.0 ± 6.6	–	–	–	0.805^T^
Support phase – less affected/healthy limb [%]	62.5 ± 6.6	62.4 ± 5.8	–	–	–	0.987^T^
Swing phase overall [%]	36.8 ± 6.4	36.3 ± 6.1	36.0 ± 6.5	0.741^T^	0.824^U^	0.777^W^
Swing phase – affected limb [%]	36.9 ± 6.4	36.4 ± 6.4	–	–	–	0.667^T^
Swing phase – less affected/healthy limb [%]	36.6 ± 6.4	36.3 ± 6.3	–	–	–	0.748^T^
Single support overall [%]	35.7 ± 5.4	35.2 ± 5.7	35.4 ± 6.6	0.980^U^	0.980^U^	0.683^W^
Single support – affected limb [%]	35.1 ± 6.4	34.9 ± 5.7	–	–	–	0.776^W^
Swing phase – less affected/healthy limb [%]	36.3 ± 5.6	35.6 ± 6.3	–	–	–	0.605^W^
Double support [%]	29.4 ± 10.7	31.1 ± 9.9	29.7 ± 13.3	1.000^T^	0.824^U^	0.162^W^
Step length overall [cm]	52.6 ± 12.5	53.3 ± 13.3	72.8 ± 18.5	0.008^T^	0.006^U^	0.690^W^
Step length - affected limb [cm]	54.2 ± 13.6	53.0 ± 13.2	–	–	–	0.265^W^
Step length – less affected/healthy limb [cm]	52.9 ± 13.9	53.5 ± 13.6	–	–	–	0.670^W^
Walking speed [m/s]	0.5 ± 0.1	0.5 ± 0.2	0.6 ± 0.09	0.155^U^	0.141^U^	0.916^W^
Cadence [number of steps/min]	63.2 ± 21.7	63.9 ± 23.5	53.0 ± 21.1	0.245^T^	0.230^T^	0.664^T^
Gait cycle [s]	1.8 ± 0.5	1.8 ± 0.5	2.0 ± 0.5	0.389^T^	0.570^U^	0.469 W

Tests for independent variables (comparing I vs. III and II vs. II): Student’s t-test: T, and Mann-Whitney U test: U

Tests for dependent variables (comparing II vs. II): Student’s t-test: T, and Wilcoxon test: W

*PAD* Peripheral Artery Disease

Comparing the gait pattern of the patient group before and after the appearance of intermittent claudication, we did not observe any statistically significant differences. However, after the appearance of claudication symptom, patients with PAD tended to shorten the step length of the affected limb. However, the difference was not statistically significant.

## Discussion

The results of this study partially correspond to the results obtained by Gommans et al. It should be pointed out that Gommans’s study is the only one published thus far which has used the optical gait analysis system Optogait to assess selected spatiotemporal parameters of gait in patients with PAD. Gommans et al. found that the step length was shorter in patients with PAD, which corroborates the results of our study. Moreover, compared to the control group, the speed and cadence of gait in PAD patients were lower by 27% and 11%, respectively, while the stance phase and double support phase were longer. As for the stance phase, the appearance of ischemic pain in the lower limbs shortened the propulsion phase by 14% and extended the flat foot phase by 17% in patients with PAD. What the two studies have in common is the fact that they included patients with symptoms in one and both lower limbs and that the patients walked on the treadmill with their preferred speed. The preferred gait speed in the study by Gommans et al. was found to be almost twice as high as that observed in our study (3.3 km/h vs. 1.8 km/h). The patients’ ability to walk faster might have stemmed from the shorter disease duration (16 months vs. 72 months). What is more, in the study by Gommans et al., the gait pattern analysis was performed when the ischemic pain reached the intensity described by the patients as “between moderate and intense” [26]. It might be assumed that these methodological differences explain why adaptive changes in the gait of patients with PAD were more intense than those observed in our study.

The results obtained are partially compatible with the findings of McCully et al., who also observed a shorter step length in PAD patients. However, contrary to our findings, they observed this abnormality only after the appearance of intermittent claudication symptoms. Moreover, in their research, the ischemic pain in the lower limbs caused a decrease in the patients’ walking speed, which was not observed in the present study. This difference between the two studies may stem from the use of different measurement devices. To measure the spatiotemporal parameters of gait, McCully used the Gait Mat system, which is a high-resolution, advanced floor mat with built-in sensors to capture plantar pressure and forces [19]. In our research, the analysis of the gait pattern was performed on a treadmill with the use of the optical Optogait system. Both methods of assessing the gait pattern are considered reliable, objective, and consistent. In order to compare the results of the two studies in a more precise way, gait pattern analysis with the use of the Optogait system should be repeated on a flat surface, with several-meter-long bars placed parallel on the walking course.

McCully’s results are in line with those obtained by Gardner, who analyzed the spatiotemporal parameters of gait with the use of a similar measurement device (GAITrite system) [7, 8]. Gardner assessed the gait pattern of PAD patients both during normal walking with a preferred speed and during a quick march. Like McCully, Gardner did not observe any abnormalities of gait during pain-free conditions. The appearance of intermittent claudication symptoms automatically altered the gait pattern. During normal walking with the preferred speed, the ischemic pain in the lower limbs caused a decrease in the speed and cadence of gait. During a quick march, the patients tended to slow down their gait, take shorter steps, and extend the double support phase. In comparison to the healthy limb, the affected limb remained longer in the swing phase and shorter in the stance phase. The difference between the results of our study and those obtained by Gardner may stem from the fact that different populations of patients were included in both studies. The average age of the participants of Gardner’s study was 71 years, while in our research, it was 64.2 ± 5.7 years. Moreover, Gardner included patients suffering from diabetes in his research, whereas in our study, diabetes was one of the exclusion criteria. It is believed that diabetic patients walk with a lower speed, take shorter and wider steps, and present a longer phase of double support in the gait compared to the healthy population [27, 28]. Therefore, it is impossible to verify to what extent the gait asymmetry of Gardner’s research participants was caused by lower limb ischaemia vs. diabetes and its neuropathic consequences.

Slightly different results are reported by Chen et al., who observed gait abnormalities in PAD patients both during pain-free and pain conditions. Their complex assessment of gait included the analysis of kinetic and kinematic values. Compared to the control group, patients with PAD walked with a lower speed and made shorter steps during pain-free conditions. They also presented an increased cadence of gait and an elongated duration of the double support phase. The ischemic pain in the lower limbs intensified the existing speed and step length abnormalities of gait [12].

On the other hand, Scherer’s research indicates that, despite the limited walking distance and lower quality of life, patients with PAD do not show any abnormalities of the gait pattern. Scherer compared the results of the patient group with the results of participants without PAD, who formed the control group. Although PAD patients obtained worse results in the walking test and in the SF-36 questionnaire, no differences in the gait parameters were observed. The assessed parameters included step length, cadence, and foot rotation angle both during walking with a preferred speed and during a quick march. It should be noted that intermittent claudication symptoms were not induced by the walking test in any of the patients. The assessment was performed only during pain-free conditions [29].

Different results obtained by the authors mentioned above seem to be the result of using different measurement devices. Although all these authors used objective methods of gait analysis, one needs to be aware of the differences that affect the quality of the assessment: methods of obtaining data, the walking surface (floor, treadmill), the positioning of markers, and the experience of the research team. The Optogait system, although a relatively new technology, is considered a credible and reliable method of analyzing gait both in physiological and pathological conditions [21–23, 30]. In order to fully exploit the potential of the measurement device applied, the gait analysis ought to be performed on a flat surface, without using the treadmill.

It should also be noted that the majority of authors do not report on the intensity of ischemic pain recorded during the gait pattern analysis. In the present study, the analysis of the gait pattern was performed when the ischemic pain reached moderate intensity, assessed by the patients as level 4 on a 5-point claudication scale. Throughout the duration of the tests, we avoided inducing the maximum ischemic pain, which is always a source of great discomfort to patients and activates an inflammation cascade. It may be presumed that with severe ischemic pain, the gait pattern is disturbed in a much more dramatic way than the gait pattern observed in patients experiencing mild/moderate pain.

The main limitations of the present study included its small population of participants and the inclusion of patients with symptoms in one and both lower limbs. Both limitations resulted from the extensive list of exclusion criteria. Another potential limitation of the study is that it assessed only selected spaciotemporal gait parameters, which do not reflect the entirety of the description of gait. Further research is required to investigate the subject in greater detail.

An important asset of this research was the homogeneity of the group of participants as regards the walking distance. Participants included in the study suffered from stage IIa of the disease according to the Fontaine classification. To standardize the functional condition of patients, the research excluded patients with a critical lower limb ischemia, patients without any symptoms of intermittent claudication, and patients for whom the walking distance was < 200 m. Another merit of the present research is the assessment of multiple spatiotemporal parameters of gait. Besides gait speed and step length, which are among the most frequently assessed gait parameters, we also analyzed the proportional share of particular phases of the gait cycle.

## Conclusions

The gait pattern of PAD patients is abnormal and characterized by a shorter step length. The appearance of intermittent claudication symptoms during the walking tests does not intensify the existing abnormalities or contribute to the formation of any new abnormalities of gait.

## Abbreviations

ABI: Ankle Brachial Index
PAD: Peripheral Artery Disease
WIQ: Walking Impairment Questionnaire
